# Dengue NS5 modulates expression of miR‐590 to regulate ubiquitin‐specific peptidase 42 in human microglia

**DOI:** 10.1096/fba.2018-00047

**Published:** 2019-03-12

**Authors:** Ritu Mishra, Vikas Sood, Akhil C. Banerjea

**Affiliations:** ^1^ Laboratory of Virology National Institute of Immunology New Delhi India; ^2^ Jamia Hamdard, deemed University New Delhi India

**Keywords:** CNS, Dengue, Dengue virus neuroinflammation, microRNA, miR‐590, NS5, TRAF6, USP42

## Abstract

Dengue virus (DENV), a member of Flaviviridae family, has become neurovirulent in humans after rapid geographical expansion. Host proteasomal machinery contains both ubiquitin ligases as well as deubiquitinases (DUBs), known to influence key cellular and biological functions. MicroRNA‐mediated modulations of DUBs in case of DENV infections have not been explored yet. DENV propagation, MiRNA overexpression, miRNA knockdown, transfection, RT‐PCR, luciferase assay, and western blotting have been used in this study to establish the interaction of miR‐590 and USP42. DENV infection in human microglial cells resulted in downregulation of host DUB‐USP42 in a dose‐dependent manner and DENV‐NS5 gene alone was found to be sufficient for this downregulation. miR‐590 was upregulated upon NS5 overexpression in a dose‐dependent manner. Downregulation of USP42 was observed with miR‐590 overexpression. The specificity of this regulation was confirmed by miR‐590 mimic and anti‐miR transfections in microglial cells. miR‐590 overexpression and knockdown affected the expression level of TRAF6 in indirect manner in microglial cells. The luciferase assay demonstrated the direct regulatory interaction between miR‐590 and 3’UTR of USP42. These findings establish that DENV‐NS5 protein can potentially modulate the host deubiquitinase protein USP42 expression via altering cellular miR‐590 levels in human microglial cells.

**Abbreviations**
AGO2: argounaute 2
CHME3: human microglial cell line
CMV: cytomegalovirus
CNS: central nervous system
CSF: cerebrospinal fluid
DENV: dengue virus
DHF: dengue hemorrhagic fever
DSS: dengue shock syndrome
DUBs: deubiquitinases
HCV: hepatitis C virus
HSV: herpes viral encephalitis
JEV: Japanese encephalitis virus
MVEV: Murray valley encephalitis virus
RIPA: radioimmunoprecipitation assay buffer
ROS: reactive oxygen species
TBST: tris‐buffered saline with Tween
THP‐1: human monocytic cell line
TRAF6: TNF receptor‐associated factor 6
UPS: ubiquitin‐proteasome system
USP42: ubiquitin specific peptidase 42
WNV: West Nile virus

## 1 INTRODUCTION

Dengue fever caused by dengue virus (DENV) is one of the most important mosquito‐borne viral disease. The world is experiencing a remarkable increase in incidences of dengue fever.^1^ As per World Health Organization (WHO) database, the global incidences of dengue fever have grown massively in recent decades. About half of the world's population is now at risk, because of unpredictable climatic changes. DENV spreads through an arthropod vector in a vicious cycle of human‐*Aedes aegypti*  mosquito‐human cycle.

DENV infection causes life‐threatening dengue hemorrhagic fever/dengue shock syndrome (DHF/DSS). Vascular hyperpermeability and plasma leakage are two major hallmarks of DHF/DSS. The mechanisms behind these pathogenic changes are still ambiguous which makes any effective therapy against DHF/DSS difficult.^2^ DENV is a member of family Flaviviridae; it is a positive‐sense RNA virus.^3^ Principally, four serotypes of DENV (DENV1, DENV2, DENV3, and DENV4) are distributed^4^ which predisposes over a third of the world's population (nearly 390 million) at grave risk of infections.^3^, ^5^ DENV is an enveloped virus which contains ∼11 kb single‐stranded RNA genome, a single open reading frame [ORF] codes for three structural proteins [capsid, prM, and envelope] as well as seven nonstructural proteins (NS1, NS2A, NS2B, NS3, NS4A, NS4B, and NS5).^4^

In advanced stages of disease, dengue patients are reported to exhibit many neurological complications like dengue encephalopathy, encephalitis, neuromuscular, and neuro‐ophthalmic complications.^6^ The exact causal factors and specific brain cell population for causing these neurological abnormalities are not very clear yet. However, activated microglial cells have been demonstrated in DENV‐infected brain tissues.^7^ Microglia are the brain‐resident macrophages which carries out multiple innate immune responses within the central nervous system (CNS) and clear out the residuals of any nearby damaged tissues.^8^ Recently, DENV has been shown to infect microglial cells and cause the acute microglial inflammation suggesting their critical role in development of encephalitis.^9^

Viral genes, proteins, and microparticles have been detected in the brains of fatal stage dengue patients^10^ as well as in experimentally infected mice brain.^11^, ^12^ The actual target of DENV‐infected cells and their bystander effects in terms of neurotoxicity and brain dysfunction has not been understood very well. Although activated microglia have been observed inside DENV‐infected brains; the molecular signaling behind this DENV‐mediated microglia modulation has not been dissected.^7^

MicroRNAs have been found circulating in nearly all body fluids. Like many other viral infections, DENV is expected to modulate host cellular microRNA profile for its own benefits. Several studies related to microRNA profiling upon dengue infection have been published,^4^, ^13^, ^14^, ^15^ which indicate its association with DENV pathogenesis. The role of miR‐146a, Let‐7e, miR‐3614‐5p, miR‐484, miR‐744, and several others are reported to influence DENV replication in different blood cell types.^13^, ^16^, ^17^, ^18^ However, none of study so far has attempted to look into the impact of DENV on microglial microRNA profile.

Human microglia, astrocytes, and brain endothelial cells are well‐documented to respond via altered cellular microRNA profile when challenged with viral proteins like HIV‐1 Tat.^19^, ^20^ Another prevalent *Flavivirus*, Japanese encephalitis virus (JEV) is known to perturb microglial microRNA profiles upon infection.^21^, ^22^, ^23^ However, contribution of microRNAs in DENV‐induced neuroinflammation is poorly understood.

Ubiquitin‐proteasome system (UPS) regulates protein turnover in cells. UPS system contains basically three principle components: the proteasome holoenzymes, multiple ubiquitin ligases, and several kind of deubiquitinating enzymes (DUBs).^24^ Many eukaryotic viruses have been recognized in influencing host protein ubiquitination machinery to facilitate their life cycle.^5^, ^25^ Similarly, deubiquitination phenomenon is also exploited by many viruses to accomplish their life cycle and manifest all facets of their pathogenesis.^26^, ^27^, ^28^ In our previous reports, we have established that one of the DUBs, USP7, stabilizes HIV‐1 Tat protein through its deubiquitination which in turn leads to enhance viral replication of HIV‐1.^25^

Ubiquitin‐specific protease 42 (USP42) is a deubiquitinating enzyme (DUB) which is widely expressed in multiple human tissues.^29^ USP42 is well‐known to target p53 and stabilizes p53 in response to stress.^30^USP42 colocalizes with RNA polymerase II (RNA Pol II) in nucleus, stays bound to histone H2B and deubiquitinate H2B. The decrease in expression level of USP42 regulates H2B ubiquitylation which suppresses both basal and induced transcription at many promoters.^30^ Studies on impact of DENV infection on host proteasomal machinery is still in its infancy and particularly regulation of proteasomal machinery by small RNAs are not explored yet. In this study, we have tried to find the nature of cross‐talk between the host deubiquitination machinery and microRNA regulatory pathway in DENV infection. DENV pathogenesis is known to predominantly influence the host inflammatory pathways which create a massive cytokine storm resulting in vascular hyperpermeability and plasma leakage. Dysregulation of microRNAs during DENV infection has earlier been reported. However, most of these studies are large‐scale screenings or arrays. USP42 profoundly influences the basal transcription level of cells and therefore is an attractive target for modulating host transcription profile. The role of USP42 or pathways that regulate USP42 expression levels/activity in case of DENV infection has not been addressed earlier.

In this study, we have investigated the functional implications of DENV infection and NS5 overexpression in human microglial cells. We have shown the role of microRNA perturbation in regulating USP42 levels and how it might affect inflammatory pathways in human microglia.

## 2 MATERIALS AND METHODS

### 2.1 Virus propagation

Dengue virus serotype 2 (DENV2/DV2) New Guinea‐C strain, NGS‐C, was expanded in C6/36 cells, cultured in Leibovitz's, L‐15 media (#11415‐049, Gibco‐Life technologies, Carlsbad, CA, USA), supplemented with 10% FBS and standard 1X concentrations of penicillin‐streptomycin‐glutamine (#A002A, Himedia, Mumbai, Maharashtra, India). C6/36 is a mosquito cell line (*Aedes albopictus*) which is non‐anchorage dependent. It was grown at 28°C temperature, without gaseous supplementation of CO_2_, and these conditions were maintained in an incubator throughout viral propagation. For propagation, 0.1 MOI of DENV were used to infect C6/36 cell and infected cells were maintained for more than 3 weeks. Following dengue infection, these cells do not undergo apoptosis and continues to grow and shed DENV. Therefore, following infection, on every third day, cell culture supernatants were collected, filtered by 0.22‐µm filters, centrifuged at 14 000 rpm and stored at −80°C. DENVs were quantified in the supernatant by a standard viral plaque assay.

### 2.2 Microglial cell culture/DENV infection

Human microglial cell line (CHME3) and HEK‐293T cells were grown in DMEM (#AL007A, Himedia, India), supplemented with 10% fetal bovine serum (#RM1112, Himedia) and 100 U of antibiotic/antimycotic solution (#A002A, Himedia). All the cells were incubated at 37°C in a humidified chamber with a constant flow of 5% CO_2_. Microglial cells were given 4 hours of serum starvation before DENV infection to enhance the affinity and viral uptake by cells. Cells were infected at different MOI (0.5, 1, 2, 3, and 4) as per experimental design as described in each experiments. After 2 hours of infection, cells were washed and were supplemented with complete media with 10% serum and antibiotic. Infected microglial cells were harvested after 24 and 48 hours as indicated in the experiments.

### 2.3 RNA isolation and microrna assay

RNA isolation was done with a miRNeasy Mini Kit (217004; Qiagen Inc, Hilden, Germany). The cDNA synthesis for all miRNAs was performed using miRNA‐specific primers and a commercial RT kit (#4366596; TaqMan Reverse Transcription Kit; Applied Biosystems, Foster City, CA, USA). Reverse transcription of microRNA was done with the following settings: 16°C for 30 minutes, 42°C for 30 minutes, and 85°C for 5 minutes. miRNA assays for miR‐590‐3p and RNU24 (small RNA control) were done using qPCR with miRNA‐specific Taqman probes and a master mix (#4324018; Universal PCR Master Mix; Applied Biosystems). PCR settings of 95°C for 10 minutes, followed by 40 cycles of 95°C for 15 seconds and 60°C for 60 seconds were used in a thermal cycler (#ABI 7500 fast Real Time PCR cycler; Applied Biosystems).

### 2.4 Cell lysates and western blot analysis/antibodies

RIPA buffer (150 mmol/L NaCl, 50 mmol/L Tris. HCL pH 7.0, 1% NP‐40, 0.5% sodium deoxycholate, 0.1% SDS, and 1X protease inhibitor cocktail (#S8820, Sigma‐Aldrich, St. Louis, MO, USA) was used to lyse the cells. Protein concentrations were estimated via a Bradford assay (#500‐0006; Bio‐Rad Laboratories, Hercules, CA, USA). Protein samples were run in a 10% polyacrylamide gel and transferred onto a nitrocellulose membrane (#SCNJ8101XXXX101 mdI, advanced microdevices Ltd., Ambala Cantt, Haryana, India) at 100 V for 2 hours. The membranes were blocked in 5% skimmed milk powder (# GRM1254, Himedia) prepared in 1X Tris‐buffered saline with Triton X‐100 (TBST). Membranes were incubated with a primary antibody (1:1000) overnight at 4°C temperature. After three washes of 15 minutes each with TBST, secondary antibodies were applied for 1 hour. Membranes were again washed in TBST three times (15 minute each) and developed. Antibodies used in this study are USP42 (#Sc‐390604, Santacruz Biotech, India), GAPDH (#Sc‐32233, Santacruz Biotech, India), anti‐HA antibody (#M1001010, Immunotag, G‐Biosciences), anti‐His antibody (#M1001020, Immunotag, G‐Biosciences), Dicer (#D38E7, Cell Signaling Technology, Danvers, MA, USA), Drosha (#D28B1, Cell Signaling Technology), AGO2 (#C34C6, Cell Signaling Technology), TRAF6 (#D2163, Cell Signaling Technology), USP7 (#D17C6, Cell Signaling Technology), dengue‐NS1 (SAB2702308‐Sigma‐Aldrich). MG132 (#C2211, Sigma‐Aldrich), a membrane permeable proteasome inhibitor, has been applied on microglia for checking the effect of the proteasomal degradation pathway on TRAF6 degradation.

### 2.5 MicroRNA overexpression and anti‐miR transfection

Microglial cells (CHME3) were seeded into six‐well plates at nearly 60% confluency, one day before transfection. Transfection mixtures were prepared in low serum commercial medium (#31985062, Opti‐MEM, Invitrogen Corp). Cells were incubated in antibiotic‐free medium during the entire transfection procedure. MiR‐590‐3p overexpression was achieved by transfecting the customized miR‐590‐3p mimic (#Eurogenetec, India) with mature miR‐590 sequence; UAAUUUUAUGUAUAAGCUAGU taken from http://www.mirbase.org. CHME3 cells were transfected with Lipofectamine RNAimax (#13778150, Invitrogen, Life technologies, Carlsbad, CA, USA) with 100 pmol and 200 pmol of miR‐590‐3p mimic. Anti‐miR‐590‐3p (#AM12644Ambion, Life‐technologies) was used to sequester out the cellular miR‐590‐3p. Anti‐miR‐590‐3p transfection was done in similar fashion with Lipofectamine RNAimax following the prescribed protocol. After 48 hours of transfection, microglia cells were harvested for protein lysate preparation as well as RNA isolation. The expression level of target protein USP42 was checked via western blotting.

### 2.6 Luciferase reporter assay

HEK293T cells were cultured in six‐well plates and cotransfected with luciferase reporter clones of USP42‐3′UTR (#SC212166‐Origene, India) and a miR‐590‐3p mimic with the help of Lipofectamine 2000 (#11668019 Invitrogen, Life‐technologies). The USP42 3′UTR clone was commercially purchased by Origene Technologies. After 24 hours of cotransfection, HEK293T cells were harvested for luciferase assays (#E151A, E153A Luciferase Assay Kit; Promega Corp, Madison, WI, USA), performed as per instructions of the manufacturer's protocol.

### 2.7 MicroRNA prediction tools

For identifying the possible microRNAs which can target USP42 gene, various bioinformatics prediction tools were utilized. MicroRNA.org, Targetscan release 7.2, MirBase, and Pictar have been utilized to check the possibility of regulatory interaction and the scores of binding between 3’UTR of USP42 and miR‐590‐3p.

### 2.8 Statistical analysis

All results are shown as mean plus standard error of the mean from three independent experiments. qPCR results are shown as relative levels compared to controls in a miR‐590‐3p assay measured by double delta Ct (ΔΔCt) algorithms. The confidence level of significance (P values) between untreated groups and treated samples were analyzed by using the Student's *t *test and *P* ≤ 0.05 has been taken as significant.

## 3 RESULTS

### 3.1 Dengue virus (DV2) infection decreases USP42 expression in human microglia in dose‐dependent manner

**Figure 1:**
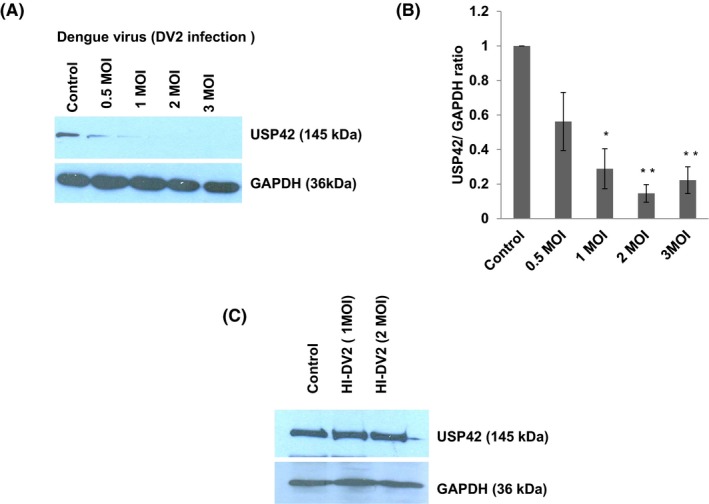
Dengue virus (DV2) infection decreases USP42 expression in human microglia in dose‐dependent manner. A, Western blot analysis of USP42 protein expression levels after DENV infection at various doses (0.5. 1, 2, and 3 MOI) on human microglial cell line CHME3. B, The graph is showing densitometry analysis of results. Image density of western blots has been normalized with GAPDH expression levels via ImageJ software. Changes in USP42 expression levels are statistically significant (*P* ≤ 0.05 and 0.005) as indicated by one * and two ** at indicated MOI of DENV infections. The experiments have been repeated three times independently and results are shown as mean ± SE (C) Western blot analysis showing no change in expression level of USP42 upon exposure with heat inactivated (HI) DENV at two different high doses (1 and 2 MOI). Heat inactivation experiments have been repeated two times to gain average value and shown as mean ± SE in the graph bars

In this study, we looked into the cellular protein expression level of USP42 to find out any modification caused by DENV in CHME3. Human microglial cells were infected with DENV2 in vitro. Microglial cells were treated with DENV in an increasing dose, ranging from 0.5, 1, 2, and 3 MOI. DENV infection caused a sharp decline in the USP42 protein expression level in a dose‐dependent manner as measured by western blotting. USP42 expression was decreased ~50% upon 0.5 MOI dengue infection. At higher MOI like 1, 2, and 3, the decrease in USP42 expression was ~75%, ~85%, and ~80% (Figure 1A,B). To ensure the specificity of DENV‐mediated reduction in USP42 expression, we also treated microglial cells with heat‐killed DENV at 1 and 2 MOI. This treatment showed no visible changes in the expression level of USP42 protein (Figure 1C), confirming the pathogenic effect of DENVs on USP42 expression levels in microglial cells.

### 3.2 Dengue NS5 protein regulates the USP42 expression level

**Figure 2:**
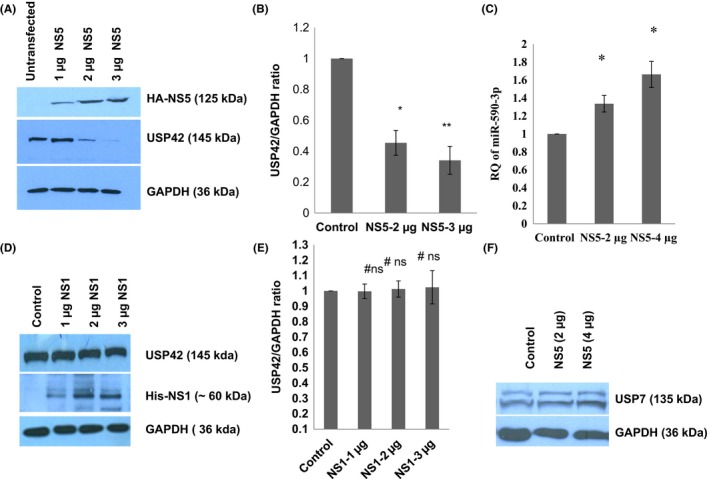
Dengue NS5 protein downregulate USP42 expression via increasing miR‐590 level. A, Western blot analysis of USP42 protein expression level after DENV‐NS5 overexpression (1, 2 and 3 µg) in human microglial cells. B, The graph bar is displaying densitometry analysis of western blot images. The graph bars are representative of three independent experiments, showing decrease in USP42 expression upon increasing doses of DENV‐NS5 overexpression. C, The graph bar is showing the relative change in miR‐590‐3p expression level upon overexpression of increasing doses of DENV‐NS5 plasmids in human microglial cells. Microglial cells were harvested for RNA analysis after 24 h of DENV‐NS5 plasmids overexpression. Relative quantification of miR‐590‐3p was done through qPCR by using human miR‐590‐3p‐specific TaqMan assay. Small RNA, RNU24, has been used as a normalizer and results are shown as fold change, compared to control. Changes in the expression levels of miR‐590‐3p are statistically significant (*P* ≤ 0.05 has been indicated by single * and *P* ≤ 0.005 by double ** asterisk symbols). D, Western blot analysis showing no significant changes in USP42 expression level upon DENV‐NS1 overexpression in increasing doses. E, The graph bars are representative of changes in USP42 expression levels after DENV‐NS1 overexpression; no significant changes as indicated by #ns. F, Western blot image showing insignificant changes in cellular USP7 expression level after DENV‐NS5 transfection in microglia. All the experiments have been repeated three times independently and results are shown as mean ± SE

Dengue virus genome contains total 10 proteins. To delineate the real effectors of depleted USP42 expression upon DENV infection, we overexpressed the HA‐tagged dengue NS5 protein for 24 hours in CHME3 cells and checked the USP42 expression levels. We used different doses of dengue NS5 plasmids for transfection to establish the cause and effect relationship of NS5 protein over USP42 expression level. We transfected 1 µg, 2 µg, and 3 µg of HA‐tagged NS5 plasmid in microglial cells and could observe the reduction in the USP42 expression level around 50% and ~70% upon 2 µg and 3 µg of NS5 transfection (Figure 2A,B). In order to confirm this, USP42 reduction as a NS5‐specific effect, we checked another potent dengue virus protein NS1 and investigated its role in modification of USP42 expression. Using the indicated concentration of NS1 plasmid transfection (1, 2, and 3 µg), the USP42 expression level remained largely unchanged (Figure 2D,E). Extending our investigations, we also checked if dengue NS5 could impact some other DUBs. Upon dengue NS5 overexpression, another important DUB the USP7 expression level was also checked. There was no significant change in the cellular expression level of USP7 (Figure 2F), while we observed a drastic downregulation of USP42 (Figure 2A,B). This led us to move further with NS5 effect on the USP42 expression level.

### 3.3 Dengue NS5 regulates the miR‐590 expression level

To dissect the cause of USP42 downregulation upon dengue NS5 overexpression, we checked whether USP42 gene sequence has strong binding sites for any relevant microRNA. Bioinformatics prediction tools suggested a strong binding site for miR‐590‐3p in 3’UTR region as displayed in Figure 5A. We checked the cellular miR‐590‐3p expression level upon dengue NS5 overexpression in CHME3 cells via a TaqMan microRNA assay and observed significant increase in the cellular expression level of miR‐590‐3p. Upon 2 µg and 3 µg of NS5 plasmid transfections in CHME3 cells, the miR‐590‐3p expression level was increased by ~1.5‐fold and ~1.9‐fold as compared to control (Figure 2C).

### 3.4 Dengue virus infection and NS5 overexpression does not alter miRNA biogenesis machinery

**Figure 3:**
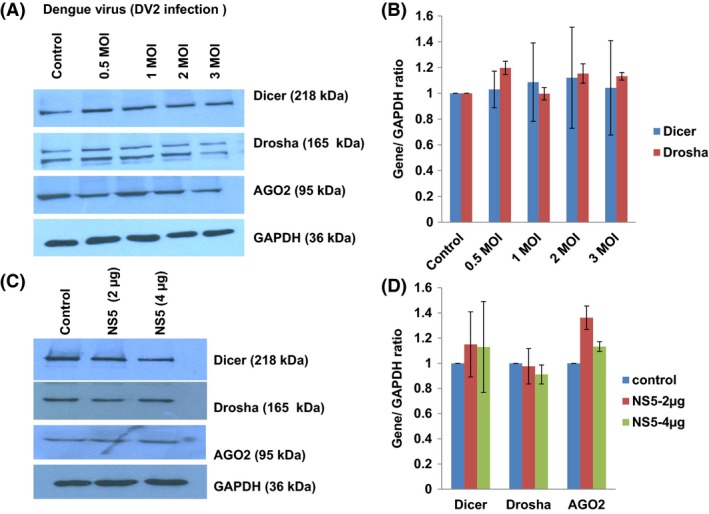
Dengue virus infection and NS5 overexpression does not alter miRNA Biogenesis machinery. A, Westren blot analysis showing the changes in cellular expression levels of major proteins involved in host miRNA biogenesis. CHME3 cells were infected with increasing doses (0.5, 1, 2, and 3 MOI) of DENV for 24 h, and cells were harvested, lysed in RIPA buffer, and checked for major proteins (Drosha, Dicer, and Ago2). B, The graph bars are representing the densitometry quantitation of Drosha, Dicer, and Ago2 protein levels; as normalized against image density of GAPDH by using ImageJ software. C, Western blots analysis of Dicer, Drosha, and AGO2 proteins upon DENV‐NS5 overexpression in human microglial cells. Microglial cells were transfected with two increasing doses (2 µg and 4 µg) of DENV‐NS5 plasmids. Upon 24 h of transfection, microglial cells were lysed in RIPA buffer and the expression level of indicated proteins were checked. D, The graph bars showing average changes in Dicer, Drosha, and AGO2 expression levels as quantified by densitometry analysis via ImageJ software. No significant changes were visible in any of indicated microRNA Biogenesis machinery proteins. Results shown here are representative of two independent biological repeats and expressed here as mean ± SE. No significant change as indicated by (*P* ≥ 0.05)

Having observed an enhanced cellular level of miR‐590‐3p upon dengue NS5 overexpression in microglial cells, we suspected an alteration of biogenesis step of microRNA. We checked the protein expression level of Dicer, Drosha, and AGO2 (which majorly constitute the microRNA Biogenesis machinery) upon dengue infection and NS5 overexpression. We could not observe any significant modulation in these protein expression levels (Figure 3A‐D). This led us to conclude that DENV infection or its one of the largest protein, NS5, does not impose any global regulation upon host cell microRNA profile via altering the microRNA biogenesis machinery.

### 3.5 miR‐590‐3p overexpression downregulates the cellular USP42 expression level

**Figure 4:**
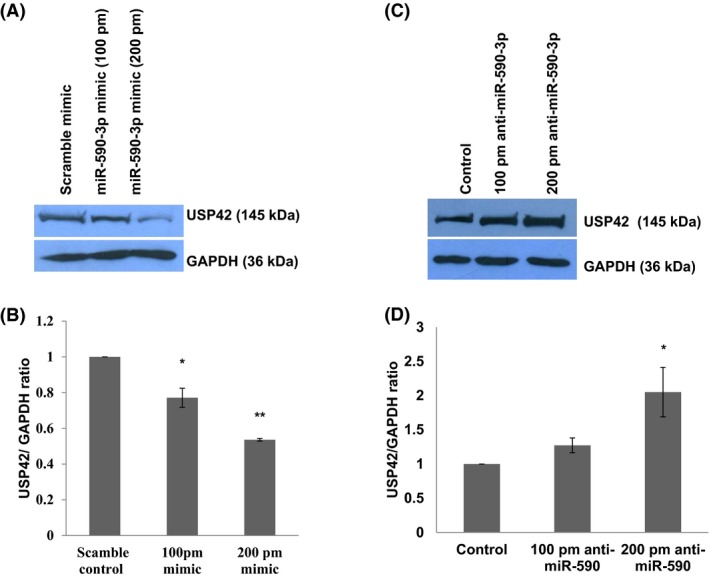
miR‐590‐3p overexpression/Anti‐miR‐590 regulate the cellular USP42 expression level. A, Cellular USP42 protein expression level after exogenous miR‐590‐3p mimics transfection. 100 picomoles and 200 picomoles miR‐590 mimic doses were applied with help of Lipofectamine RNAiMax. Lysates were prepared after 24 h and western blot analysis was done. B, The graph bars were prepared after Image densitometry; USP42 expression levels were normalized with respective GAPDH image densities. Significant changes in USP42 protein expression level indicated by (*P* ≤ 0.05 as indicated by *) in 100‐picomole transfection and (*P* ≤ 0.005 as indicated by **) in 200‐picomole miR‐590 mimic transfection. C, USP42 protein expression levels upon anti‐miR‐590 transfection in CHME3 cells. At both the doses (100 and 200 picomoles), USP42 expression levels increased in dose‐dependent manner. D, The graph bars are representing the average change in USP42 expression upon anti‐miR‐590 transfection. All the experiments have been performed as three independent biological repeats. Data are represented by mean ± SE *P* values have been determined by Student's *t* test. The level of significance is shown as (*P* ≤ 0.05 as indicated by *)

Bioinformatics analysis (MicroRNA.org, miRDB) strongly suggested the possible target site for miR‐590‐3p in 3’UTR of USP42 which needed to be confirmed at the cellular level. For confirming regulatory effect of miR‐590‐3p, we exogenously overexpressed the miR‐590‐3p in CHME3 cell. miR‐590‐3p mimics were transfected at 100 picomoles and 200 picomoles and changes in USP42 expression levels were checked through western blotting. At both concentrations of miR‐590 mimics, the USP42 expression level was decreased in a dose‐dependent fashion (~25% decrease at 100 pm and ~50% decrease at 200 pm mimic transfection) as shown in Figure 4A,B. We stretched the miR‐590‐3p mimic transfection even at 400 pm which drastically reduced the USP42 expression (almost 80%) in CHME3 cells (data not shown).

### 3.6 Anti‐miR‐590 transfection rescues the expression level of USP42

miR‐590 targets the cellular USP42 expression level in microglial cells. To further strengthen our statement, we applied miR‐590 inhibiter (here described as anti‐miR‐590) in human microglial cells. Anti‐miR‐590 was transfected at two concentrations, 100 picomoles and 200 picomoles, in microglial cells, and after 48 hours, cells were harvested to check the target USP42 protein expression levels. Cellular USP42 expression levels were increased ~50% and ~100%, respectively, as compared to control cells, as shown in Figure 4C,D. This observation indicated a rescue in the cellular USP42 expression as a result of sequestering out of cellular miR‐590.

### 3.7 miR‐590‐3p directly targets the 3’UTR of USP42

**Figure 5:**
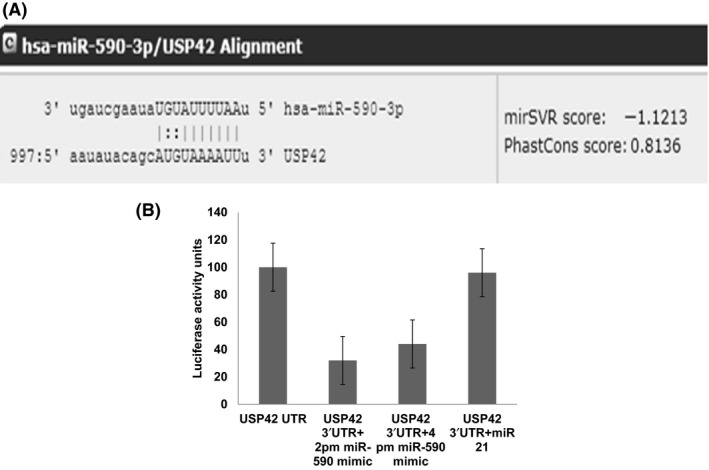
miR‐590‐3p directly targets the 3’UTR of USP42. A, A snapshot image from microRNA.org to show seed sequences in miR‐590‐3p and complementary 8‐mer binding sites in USP42 3’UTR. Image is also showing a high mirSVR score −1.1213, indicating strong free energy change of this interaction. USP42 3’UTR reporter construct were cotransfected with miR‐590 mimic and miR‐21 plasmids separately and luciferase assays were performed in the HEK293T cells. B, miR‐590 mimic transfection significantly inhibited the luciferase activity (almost 70% decreases) in USP42 3’UTR cotransfection. An irrelevant miR‐21, which does not have the complementary sequences for USP42 3’UTR could not inhibit the luciferase activity. The luciferase expression level in (only 3’UTR of USP42‐transfected HEK293T) was considered as control with 100% RLU unit and result has been shown with respect to that. Cotransfection experiment has been done with two doses (100 picomoles and 200 picomoles) of miR‐590 mimic along with 3’UTR of USP42. All the doses make a significant suppression of luciferase activity

Dengue NS5 induced the expression level of miR‐590‐3p in microglial cells. To find out the potential targets of human miR‐590‐3p, we used various online available bioinformatics tools **(**MicroRNA.org, Targetscan 7.2, Pictar, etc). These bioinformatics tools showed very strong and multiple binding sites in 3’UTR region of USP42 as shown in Figure 5A. A distinct dose‐dependent increase in the expression of miR‐590‐3p after NS5 treatment and the concordant decrease in the expression level of USP42 already suggested that the expression level of USP42 might be under regulation of miR‐590‐3p. 3’UTR of USP42 being one of the longest (1061 base pairs) also predisposes its regulation via microRNA. To confirm its posttranscriptional regulation being mediated by miR‐590‐3p, we did a luciferase assay for standard target validation. HEK293T cells were cotransfected with 3’UTR construct of USP42 (flanked with luciferase coding sequences) and miR‐590 mimic. After 24 hours, a luciferase assay showed almost ~65% reduction in luciferase expression in this experiment (Figure 5A,B). Only 3’UTR transfected cells were measured for a luciferase assay and treated as 100% control. Another irrelevant microRNA, miR‐21, was also cotransfected with 3’UTR of USP42 to emphasize the specificity of sequence‐dependent complementary binding between USP42 3’UTR and miR‐590 only. In this unrelated miR‐21 and 3’UTR of USP42 cotransfection, there was no reduction in the luciferase expression level (Figure 5B).

### 3.8 miR‐590 modulates the TRAF6 expression level in HUMAN microglial cells

**Figure 6:**
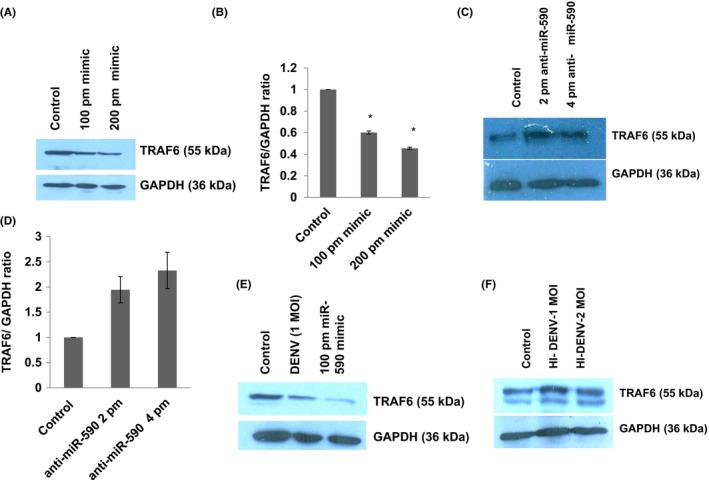
TRAF6 expression level is regulated indirectly via miR‐590 in human microglial cells. A, Western blot image showing expression level of TRAF6 protein upon miR‐590 mimic transfection at 100 pm and 200 pm. TRAF6 expression decreases in dose‐dependent manner. B, The graph bars are showing average change in TRAF6 expression levels. Image densitometry was done with ImageJ software while taking GAPDH as loading control. Experiments have been repeated two times independently. Data are represented by mean ± SE; p values have been determined by Student's *t *test. The level of significance is shown as (*P* ≤ 0.05 as indicated by *). C, Western blot image showing increase in TRAF6 expression level upon anti‐miR‐590 transfection in microglial cells at two different concentration, that is, 2 pm and 4 pm. D, The graph bars are displaying average change in TRAF6 expression levels. Image densitometry analysis was done with ImageJ software while taking GAPDH as a loading control. Experiments have been independently repeated two times. E, Western blot analysis showing the impact of DENV virus infection and miR‐590 mimic transfection upon TRAF6 expression level. F, Western blot analysis showing unchanged TRAF6 expression level after infection with heat inactivated DENV at two doses. Experiments have been repeated two times independently and representative images are shown in the result

**Figure 7:**
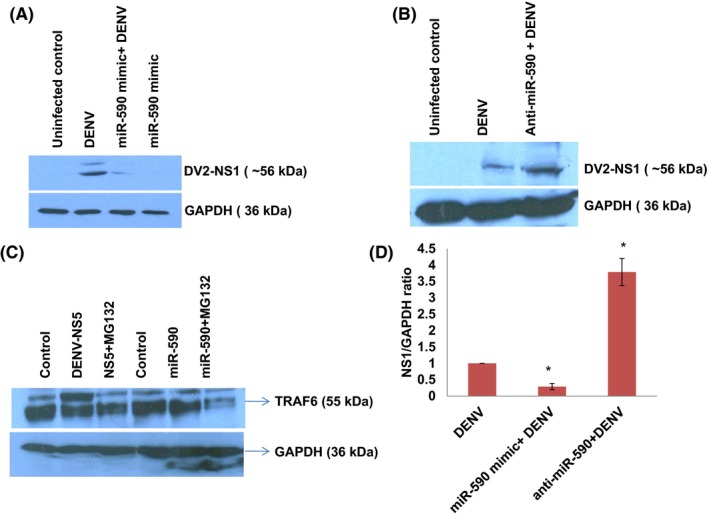
miR‐590‐3p influences DENV replication. A, Western blot image showing decreased DENV‐NS1 level when microglial cells are pretreated with miR‐590 mimic. Microglial cells were transfected with 100 pm of miR‐590 mimic, after 12 h of mimic transfection, DENV was infected at 1 MOI. After 24 h of virus infection, cells were harvested, lysed, and analyzed by western blotting. B, Western blot image showing the level of DENV‐NS1 as an indicator of DENV replication upon anti‐miR‐590 transfection. Microglia were transfected with 100 pm of anti‐miR‐590, followed by DENV infection at 1 MOI. After 24 h of virus infection, cells were harvested, lysed, and analyzed by western blotting. C, Western blot image showing impact of proteasomal inhibitor MG132 on TRAF6 stabilization. Cells were transfected with DENV‐NS5 and miR‐590 mimics with lipofectamine RNAiMax reagent. After 12 h, MG132 was given to cells at 20 µM final concentration. After 8 h of the inhibitor treatment, cells were harvested and analyzed via western blotting for the cellular level of TRAF6. GAPDH protein levels have been taken as a loading control. D, The bar graph is showing the DENV‐NS1 level comparison in contrasting miR‐590 and anti‐miR‐590‐transfected microglial cells

TRAF6 is an E3 ligase protein which consists of a RING domain and functions in association with Ubc13/Uev1A to synthesize a unique polyubiquitin chains consisting of lysine‐63 (K63) of ubiquitin.^31^ Considering the critical importance of TRAF6 in DENV life cycle,^18^, ^32^ we were interested to check the impact of miR‐590 on the TRAF6 expression level. TRAF6 expression levels significantly went down upon miR‐590 mimic transfection in human microglial cell (Figure 6A,B). As expected, anti‐miR‐590 transfection was able to upregulate the TRAF6 expression level in microglial cells (Figure 6C,D). We have also checked whether DENV infection can reduce the TRAF6 expression level in microglial cells since it was earlier shown to be downregulated in other cell lines (primary human monocytes and THP‐1 cells) upon DENV infection.^18^, ^32^ The TRAF6 expression level was downregulated upon 1 MOI of DENV infection in microglial cells (Figure 6E). This dowregulation was abolished when we infected microglial cells with heat killed DENV (Figure 6F). To check if the decrease of TRAF6 is due to the enhancement of protein degradation or the decrease of the transcripts, we did the proteasomal inhibition assay. CHME3 cells were transfected with DENV‐NS5 and miR590 mimic as in previous experiments. After 12 hours of transfections, cells were treated with 20 µM of MG132 for next 8 hours. Afterward, cells were harvested and checked for cellular TRAF6 expression levels. The TRAF6 expression level was decreased in DENV‐NS5–transfected cells as well as miR‐590 mimic‐transfected cells. However, in MG132‐treated lane (proteasomal degradation pathway blocked), TRAF6 levels were not stabilized or enhanced (Figure 7C). This result suggested that downregulation of TRAF6 is not via proteasomal degradation. It raises the possibility that DENV‐NS5 and miR‐590 induced dowregulation of TRAF6 might be under the influence of transcriptional/posttranscriptional regulation (microRNA‐mediated translation blockage).

### 3.9 miR‐590‐3p influences DENV replication

**Figure 8:**
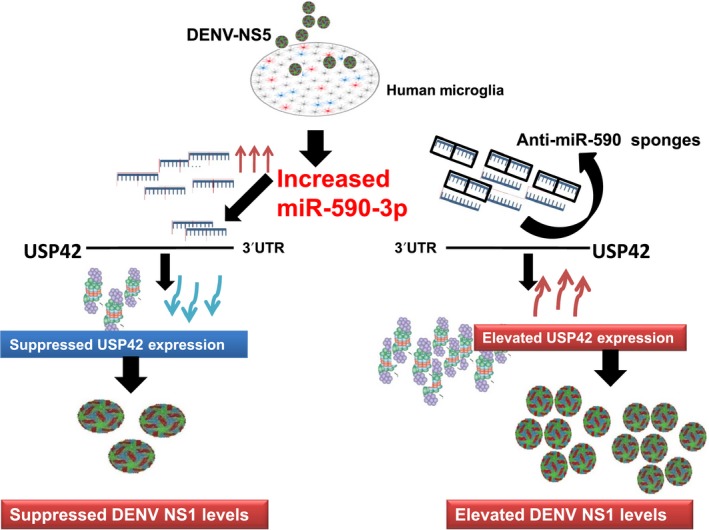
Schematic for DENV‐NS5‐mediated regulation of USP42 via miR‐590 in human microglia. A working model for DENV‐NS5 protein‐mediated regulation of a host deubiquitinase USP42, via elevating host miR‐590‐3p in human microglial cells. miR‐590‐3p has strong complementary binding site within its seed sequences for 3’UTR of USP42 mRNA. The enhanced expression level of miR‐590 by DENV‐NS5 overexpression helps to tie down the cellular USP42 expression level which in turn affect the DENV replication cycle in human microglial cells

The ultimate impact of the molecular changes brought upon by perturbed cellular level of miR‐590 and their effect on viral replication state was measured by estimation of one of the major dengue protein NS1. As shown in Figure 7A,B,D, DENV NS1 levels were decreased when microglia were pretreated with miR‐590 mimics. However, when microglial cells were treated with anti‐miR‐590, NS1 levels were found increased. These results suggest us that miR‐590 has a role in regulating dengue replication and thus its life cycle; however, the detail mechanism of this outcome would be explored in our upcoming studies (Figure 8).

## 4 DISCUSSION

Viral infections of the CNS are often caused by RNA viruses and neurological manifestation are quite common in JEV, ZIKA virus infections.^33^ Viral encephalitis is the most common manifestation of virus infection in brain. Japanese encephalitis virus (JEV), West Nile virus (WNV), Murray valley encephalitis virus (MVEV), herpes simplex virus (HSV), human immunodeficiency virus (HIV), cytomegalovirus (CMV), hepatitis C virus (HCV), and several other viruses are known to cause varying degree of neurological abnormalities.^34^

In this study, we have demonstrated, how DENV NS5 protein alters the host cellular microRNA, miR‐590‐3p, in microglial cells which in turn downregulate USP42 (a deubiquitinase). We were interested in finding out the impact of DENV infection/viral protein on host deubiquitinase machinery especially in human microglial cells since microglial cells are the brain resident macrophages and confer protection to the human brain.

Human genome encodes ~95 DUBs categorized into five major families, out of which USPs (Ubiquitin specifc peptidase) are the largest family.^26^, ^35^ In a previous study, RNAi screening has listed USP42 as a regulatory factor in HCV infection.^36^ As per another screening study, it was shown to be modulated in *Vaccinia* virus infection.^37^ In a recent study, USP13 has been reported to stabilize STAT1 and influencing type I interferon pathway in dengue infection.^38^ However, most of these studies were large‐scale screening studies/array. The role of USP42 or what might regulate USP42 expression levels/activity in case of viral infection has not been investigated at all. It is also recently reported that DUBs activity can be reversibly inhibited by oxidation mediated by reactive oxygen species (ROS).^39^

Our results demonstrated that dengue viral infection as well as dengue NS5 overexpression were able to downregulate the endogenous USP42 expression level in human microglial cells. To find the molecular signaling pathway for this downregulation of USP42 expression, we took help of bioinformatics prediction tools. We used programs like Targetscan, MicroRNA.org, MirBase to find out potential regulatory microRNAs. All of these databases suggested strong binding sites in 3’UTR region of USP42 for seed sequences of miR‐590‐3p. We therefore thought that this miR‐590 must have some important regulatory significance in dengue pathogenesis. In general, majority of miRNAs reside inside cells but many of regulatory microRNAs are frequently found outside the cells, for example, our body fluids like serum, plasma, CSF, urine, and saliva. MicroRNAs have been reported to get packaged in microvesicles and exosomes. However, in case of freely circulating microRNAs in body fluids; they are extensively protected by RNA binding proteins.^40^, ^41^, ^42^

Since dengue NS5 is known to localize in the nucleus and influence the host transcription/splicing machinery, we investigated the functional implication of modulation of USP42. We chose USP42 in particular as this deubiquitinase is known to regulate the functions of the histone proteins in host cell via regulating the ubiquitination and deubiquitination of histones. Thus, USP42 has an important and direct role in regulation of host‐transcriptional machinery.

We could observe a specific USP42 downregulation in case of dengue NS5 overexpression but not in case of dengue NS1 overexpression (Figure 2D). This demonstrates the remarkable job compartmentalization among viral proteins. It could be spatial as well as temporal in case of different viral infections. It was reported in case of HIV‐1 infection also, where two different viral proteins (Tat and Rev) could act at different time point in HIV‐1 life cycle to complete their successful life cycle.^43^ HIV‐1 Rev could downregulate Tat protein and thus HIV‐1 transcription as whole, was shut down in later stages of viral life cycle. In our experiments, heat‐inactivated DENV was found ineffective in exerting any expression level change in USP42 (Figure 1C), which indicated that USP42 downregulation is a cellular signaling phenomenon triggered only upon successful viral entry inside host cells rather than a surface interaction phenomenon between host cell receptor and DENV envelop proteins. miR‐590‐3p levels were checked to find any regulatory role in cellular USP42 expression upon DENV NS5 overexpression. It was increased in a dose‐dependent manner (Figure 2C), strongly indicating that USP42 expression might be under influence of cellular miR‐590‐3p. Observation of miR‐590‐3p upregulation also raised a possibility that microRNA biogenesis machinery as such might be under regulation by DENV virus/NS5 protein. To rule out this scenario, we checked the expression level of major microRNA biogenesis machinery protein such as Dicer, Drosha and AGO2 protein. In both the experimental conditions either DENV virus infection or DENV NS5 overexpression, no substantial change was observed in Dicer, Drosha, or AGO2 protein expression levels (Figure 3). It helped us to conclude that DENV does influence selective microRNA expression to modulate its own specific cellular target and does not globally divert the microRNA biogenesis machinery. This has also been reported in case of HIV‐1 Tat protein impact on microRNA biogenesis machinery, where it does not play much with cellular expression level of Dicer, Drosha, or AGO2 protein.^19^, ^20^ In a recent report, DENV was shown to utilize host microRNA biogenesis machinery for generating its very own viral microRNA. Argonaute 2 was found mainly involved in DENV–vsRNA‐5 biogenesis.^44^ This explains why DENV would not be inclined toward decreasing or hampering any host microRNA biogenesis machinery. For confirming the regulation of USP42 gene expression by miR‐590‐3p in human microglia, the standard validation techniques (overexpression, anti‐miR transfection, and luciferase assay) were followed. In both the experiments of miR‐590‐3p overexpression and anti‐miR‐590 transfection, it was tested with two doses of mimic as well as anti‐miR concentrations. As we saw (Figure 4A‐D) that the USP42 protein expression level does modulate under influence of miR‐590. This result again validated the role of miR‐590 in regulating the USP42. For concrete verification, we performed luciferase assay. In this experiment, USP42 3’UTR were cloned into a luciferase plasmid construct. In this construct, USP42 3’UTR sequence was flanked downstream of luciferase coding region. In cotransfection experiments (3’UTR+miR‐590 mimic), the luciferase expression went down upto 65%. It strongly suggested that seed sequences of miR‐590‐3p have strong binding affinity for complementary sequences in USP42 3’UTR region. MicroRNA.org also suggests that miR‐590 has more than one binding sites with different scores within 3’UTR of USP42. With irrelevant microRNA such as miR‐21, the luciferase expression was unaltered. This experiment conclusively established that USP42 3’UTR possess the complementary sequences toward seed sequences of miR‐590 and USP42 gene expression can be effectively modulated through micromanaging the cellular miR‐590 expression level. Nonetheless, this regulation of USP42 expression is highly specific since miR‐21 could not exert such regulation upon USP42 3’UTR luciferase expression.

To judge overall impact of changing expression level of cellular miR‐590 on DENV pathogenesis, we checked the expression levels of TRAF6 protein upon miR‐590 mimic and anti‐miR‐590 transfection. TRAF6 is a well‐known inducer of type I interferon pathway and have been found dampened upon DENV infection in human monocytes/THP1 cell line to facilitate DENV replication.^18^ DENV infection targets the expression level of TRAF6 via inducing the expression of miR‐146a and diminishes IFN‐β production which facilitate viral replication.^31^ TRAF6 is also recognized as a key molecule in modulating the DENV‐autophagy interaction.^18^ In our study too, DENV infection decreases the TRAF6 expression level in human microglial cells (Figure 6E). In case of heat‐inactivated DENV infection this effect disappears (Figure 6F). Exogenous overexpression of miR‐590 downregulate the cellular TRAF6 expression level in microglial cells and anti‐miR‐590 transfection increases the TRAF6 expression level (Figure 6C). Although, in bioinformatics prediction tools, the interaction between TRAF6 3’UTR and seed sequences of miR‐590 was not strongly indicated. We regard this change in the TRAF6 expression level due to some indirect effect of USP42, which we will consider for future investigation. Similarly, the DENV replication levels measured via NS1 protein have also shown a very interesting trend. NS1 levels appears to be under influence of miR‐590 (Figure 7A,B); however, we cannot comment with full confidence whether this change is due to direct regulatory effect of miR‐590 on DENV genome or through indirect cascade of immune signaling. We will put our future efforts to dissect the molecular mechanism behind these changes in DENV replication.

Considering the USP42, a deubiquitinase, with multifunctional roles, we regard this result open for many explanation. There is another aspect of miR‐590 being capable of targeting many more host genes and not just the USP42 gene expression. Keeping both these above factors in mind, the DENV NS5‐mediated upregulation of cellular miR‐590 and consequent downregulation of USP42 gene suggests us that in basal conditions; USP42 is more likely to play a multidimentional role in the modulation of host‐pathogen interaction in microglial cells that will ultimately be responsible for controlling viral replication. This is why the DENV infection and DENV NS5 overexpression in micoglial cells, both are attempting to modify the cellular USP42 level.

## 5 CONCLUSIONS AND SIGNIFICANCE

In this study, we demonstrated for the first time that DENV infection as well as DENV‐NS5 protein independently can modulate a host deubiquitinase protein, USP42, through the miR‐590‐mediated pathway in human microglial cells. The modulation of miR‐590 via mimic and antagonist of miR‐590 also impacts the TRAF6 expression level which is a major regulator of host inflammatory responses.

This piece of study is quite significant for the broad biomedical community who are pursuing various studies on viral pathogenesis. Earlier, dengue‐infected patients’ serum has displayed heavily dysregulated “fingerprint” microRNAs such as miR‐146, miR‐21, and miR‐590.^45^ The role of miR‐146 and miR‐21 are quite known in regulation of early inflammatory pathways but function of miR‐590 was not explored to pinpoint its precise role in dengue pathogenesis. Similarly, role of deubiqitinases in dengue viral pathogenesis is also poorly understood. Since magnitude of immune responses play a very critical role in dengue pathogenesis, it is very important to have a comprehensive picture of interacting immune signaling molecules and consequences of these observed interactions and cellular responses. This study provides a new angle into the host‐pathogen interplay inside human microglial cells during DENV‐associated neuroinflammation. Our study also suggests how a serum circulating microRNA such as miR‐590, which is enhanced during DENV infections, might affect the distant cellular gene expression profile and further enhance DENV pathogenesis. It presents a novel signaling cascade where dengue virus protein NS5 specifically employs miR‐590 to downregulate USP42 expression. This is the first report to our knowledge that clearly demonstrates miR‐590 mediated regulation of host proteasomal machinery.

## CONFLICT OF INTEREST

The authors had no competing conflicts of interest to disclose.

## AUTHOR CONTRIBUTIONS

R. Mishra and A.C. Banerjea conceived the idea; R. Mishra designed the study, performed all the experiments, and data analysis. V. Sood helped in generating the virus stock and plaque assay. R. Mishra and A.C. Banerjea wrote the paper.
